# HEALTH CARE UTILIZATION AND COSTS FOR HOSPITALIZED DEMENTIA PATIENTS IN CHINA

**DOI:** 10.1093/geroni/igae098.3607

**Published:** 2024-12-31

**Authors:** Zhuoer Lin, Fang Ba, Heather Allore, Xi Chen

**Affiliations:** University of Illinois Chicago, Chicago, Illinois, United States; Yale University, New Haven, Connecticut, United States; Yale University, New Haven, Connecticut, United States; Yale University, New Haven, Connecticut, United States

## Abstract

**Background:**

Dementia poses challenges to patients and healthcare systems; yet little is known about the healthcare utilization and costs for dementia patients in China and their major contributors.

**Methods:**

We identified 51,530 inpatient records of Chinese older adults who were admitted to 1,917 hospitals during 2017-2019 with a primary diagnosis of dementia. Multivariable logistic regressions estimated associations for emergency room (ER) admission and tertiary hospital admission; and linear regressions were performed to examine the determinants of length of stay (LOS), daily and total costs in Chinese Yuan (CNY). The potential contributors included age, sex, insurance type, working status, comorbidity, admission type and hospital level.

**Results:**

The sample had a median age 78 (IQR: 72-84), of whom 46.2% were females, 20.2% were admitted through an ER, and 69.0% were admitted to tertiary hospitals. LOS, daily and total costs per admission were 21 days, 948 CNY and 12,602 CNY, respectively. Increased adjusted odds of ER admission were associated with having lung diseases or lower insurance coverage. LOS was longer for older patients, females, retirees, or those with more insurance coverage or admitted to lower-level hospitals. The total costs were higher among older patients, males, patients with lung diseases, more insurance coverage, and patients admitted to ER or tertiary hospitals.

**Conclusion:**

There are large variations in inpatient care utilization and costs across dementia patients of different demographics, insurance types, and comorbidities. Our findings highlighted the importance of promoting chronic disease management and insurance coverage, as well as providing affordable and equitable dementia care.

